# Cerebral autoregulation in traumatic brain injury: ultra-low-frequency pressure reactivity index and intracranial pressure across age groups

**DOI:** 10.1186/s13054-024-04814-5

**Published:** 2024-01-23

**Authors:** Paolo Gritti, Marco Bonfanti, Rosalia Zangari, Ezio Bonanomi, Alessia Farina, Giulio Pezzetti, Isabella Pelliccioli, Luca Longhi, Maria Di Matteo, Andrea Viscone, Gabriele Lando, Gaia Cavalleri, Simonetta Gerevini, Francesco Biroli, Ferdinando Luca Lorini

**Affiliations:** 1 Department of Anesthesia and Critical Care Medicine, ASST Papa Giovanni XXIII Hospital, Bergamo, Italy; 2 FROM Research Foundation, Papa Giovanni XXIII Hospital, Bergamo, Italy; 3 Department of Neuroradiology, Papa Giovanni XXIII Hospital, Bergamo, Italy

**Keywords:** Cerebral autoregulation, Ultra-low-frequency pressure reactivity index, Pressure reactivity index, Age group, Traumatic brain injury, Intracranial pressure, Cerebrovascular reactivity, Optimal cerebral perfusion pressure

## Abstract

**Background:**

The ultra-low-frequency pressure reactivity index (UL-PRx) has been established as a surrogate method for bedside estimation of cerebral autoregulation (CA). Although this index has been shown to be a predictor of outcome in adult and pediatric patients with traumatic brain injury (TBI), a comprehensive evaluation of low sampling rate data collection (0.0033 Hz averaged over 5 min) on cerebrovascular reactivity has never been performed.

**Objective:**

To evaluate the performance and predictive power of the UL-PRx for 12-month outcome measures, alongside all International Mission for Prognosis and Analysis of Clinical Trials (IMPACT) models and in different age groups. To investigate the potential for optimal cerebral perfusion pressure (CPPopt).

**Methods:**

Demographic data, IMPACT variables, in-hospital mortality, and Glasgow Outcome Scale Extended (GOSE) at 12 months were extracted. Filtering and processing of the time series and creation of the indices (cerebral intracranial pressure (ICP), cerebral perfusion pressure (CPP), UL-PRx, and deltaCPPopt (ΔCPPopt and CPPopt-CPP)) were performed using an in-house algorithm. Physiological parameters were assessed as follows: mean index value, % time above threshold, and mean hourly dose above threshold.

**Results:**

A total of 263 TBI patients were included: pediatric (17.5% aged ≤ 16 y) and adult (60.5% aged > 16 and < 70 y and 22.0% ≥ 70 y, respectively) patients. In-hospital and 12-month mortality were 25.9% and 32.7%, respectively, and 60.0% of patients had an unfavorable outcome at 12 months (GOSE). On univariate analysis, ICP, CPP, UL-PRx, and ΔCPPopt were associated with 12-month outcomes. The cutoff of ~ 20–22 for mean ICP and of ~ 0.30 for mean UL-PRx were confirmed in all age groups, except in patients older than 70 years. Mean UL-PRx remained significantly associated with 12-month outcomes even after adjustment for IMPACT models. This association was confirmed in all age groups. UL-PRx resulted associate with CPPopt.

**Conclusions:**

The study highlights UL-PRx as a tool for assessing CA and valuable outcome predictor for TBI patients. The results emphasize the potential clinical utility of the UL-PRx and its adaptability across different age groups, even after adjustment for IMPACT models. Furthermore, the correlation between UL-PRx and CPPopt suggests the potential for more targeted treatment strategies.

*Trial registration*: ClinicalTrials.gov identifier: NCT05043545, principal investigator Paolo Gritti, date of registration 2021.08.21.

**Supplementary Information:**

The online version contains supplementary material available at 10.1186/s13054-024-04814-5.

## Introduction

Cerebral autoregulation (CA) may be impaired in patients with traumatic brain injury (TBI) and is potentially a significant risk factor for secondary injury [1, 2]. Continuous monitoring of CA is pivotal in ensuring adequate perfusion to the injured brain and provides an opportunity to tailor the optimal state of autoregulation for the patient, the optimal cerebral perfusion pressure (CPPopt) [2–4]. One of the most widely used surrogate methods for continuous bedside estimation of CA is the pressure reactivity index (PRx). It is calculated as a moving correlation coefficient between 30 consecutive 10-s average values of intracranial pressure (ICP) and mean arterial pressure (MAP) waveforms [2]. This method enables continuous bedside monitoring of CA, and when plotted against cerebral perfusion pressure (CPP), the CPPopt, can be determined [2, 4–7]. A specialized software is required for PRx calculation and assessment, and in general, cerebrovascular reactivity indices have the limitation of requiring high-frequency data sampling and export for post-acquisition processing [8, 9].

A common problem with numerous bedside monitors in the intensive care units (ICU) is their inability to export data in a period lower than 1 min. In response, researchers attempted to use minute-by-minute ICP and MAP data as a surrogate approach for estimating CA, which led to the development of an alternative algorithm, the long-PRx (L-PRx). This approach led to promising results as shown by several studies [6, 9–12].

Recently, we proposed a variant of the L-PRx derived from a 5-min average of ICP and MAP values and calculated using 30 min moving correlation window, the ultra-low-frequency pressure reactivity index (UL-PRx) [13, 14]. Although UL-PRx provides enough resolution to derive information about the state of cerebrovascular autoregulation, an application to a large cohort of adult and pediatric TBI patients and an attempt to validate it with the International Mission for Prognosis and Analysis of Clinical Trials (IMPACT) models (Core, Core + computed tomography (CT) and/or laboratory variables (Lab)) [15] were not pursued. Furthermore, it is not yet known whether such low-frequency sampling can lead to the development of a surrogate algorithm that enables determination of the CPPopt.

Regarding the comparison between ICP and derived indices in children and adults or the changes with age, the literature is limited. The general notion that children have lower ICP values than adults is not well documented, and there appear to be no validated guidelines. Consequently, the same estimated ICP reference range is used indiscriminately for child and adult, regardless of the many other documented physiological differences between children and adults [16].

The main objective of the study was to test UL-PRx for its discriminative and predictive ability regarding mortality and outcome at 12 months exploring the possibility to obtaining the CPPopt even with a low-frequency sampling data.

The second objective of this study is to explore the association between ICP and derived indices and 12 months outcomes in different age groups.

## Material and methods

Data of consecutive adult and pediatric TBI patients (0–85 years) admitted to the ICU of ASST Papa Giovanni XXIII, who required ICP monitoring during the study period from January 2013 to June 2022, were extracted. Inclusion criteria were availability of continuous intensive care monitoring of physiological data (ICP, MAP, and CPP). Demographic data, Glasgow Coma Scale (GCS) on admission, Motor Score (GCS-Motor), pupillary reactivity (bilaterally reactive, unilateral reactive, and bilateral unreactive), documented hypoxia (peripheral oxygen saturation < 90%) or hypotension (systolic pressure < 90 mmHg), radiological presentation (main characteristics of the most severe brain CT scan performed in the first 24 h of hospitalization) for the assessment of Marshall CT score [17] and the presence of traumatic subarachnoid hemorrhage (SAH) and/or extradural hematoma (EDH), serum levels of hemoglobin and glycemia detected in the emergency department, neurosurgical procedures (primary or secondary decompressive craniectomy (DC)), length of hospital and ICU stay (LOS), in-hospital mortality, and outcome at 12 months (Glasgow Outcome Scale Extended (GOSE)) [18] were considered. The outcome was dichotomized as: non-fatal (GOSE 2–8) versus fatal (GOSE 1) and favorable (GOSE 5–8) versus unfavorable (GOSE 1–4) at 12 months.

Patients with missing data, who did not meet these criteria, or for whom the monitoring time was less than 4 h were excluded. The entire process has been described in detail previously [13, 14].

### Ethics committee

The study protocol complies with the ethical guidelines of the Declaration of Helsinki and was approved by Bergamo’s ethics committee (see Declarations). The Standards for Reporting of Diagnostic Accuracy Studies Statement and Strengthening the Reporting of Observational Studies in Epidemiology were followed [19]. ClinicalTrials.gov identifier: NCT05043545.

### Patients management

Adult and pediatric TBI patients were managed in the dedicated ICUs according to the internal standardized protocol and literature guidelines [20–23].

Patients were sedated and intubated, and invasive monitoring including continuous evaluation of MAP, central venous pressure, ICP, and CPP were performed. The arterial transducer was positioned at level of the tragus, and CPP was calculated as the difference between MAP and ICP. Hyperthermia and hyponatremia were strictly avoided, and CPP was maintained within the limits defined by age and respective guidelines, using catecholamines or increasing volemia as needed [21–23]. Intracranial hypertension defined by age and respective guidelines was treated with a stepwise approach including head elevation, deep sedation and neuromuscular blockade, boluses of mannitol or hypertonic solutions, moderate hyperventilation, and cerebrospinal fluid subtraction. DC was performed when the ICP was refractory to maximal medical therapy [20–23].

### Data acquisition filtering and processing

ICP and MAP data were collected through intraparenchymal catheters probes (Codman MicroSensor ICP transducer, Codman and Shurtleff, Inc., Massachusetts, USA), generally inserted into the frontal white matter of the injured hemisphere and via radial or femoral artery catheters, respectively, and monitored continuously. CPP was calculated as the difference between MAP and ICP. Physiological measurements were sent to the database Systems (GE’s Centricity Critical Care, Chalfont St. Giles, UK) at ~ 0.0033 Hz (about an average period of 5 min) and retrospectively extracted through interactive SQL (Toad ® for SQL, Quest Software, California). The time series were manually filtered and resampled on a minute-by-minute basis using linear interpolation. Incomplete recordings lasting more than 25 min were automatically identified, and time series shorter than 60 min were disregarded. Data preparation and index calculations were performed using an in-house algorithm (MATLAB R2020a; MathWorks, Natick, MA, USA). The procedures for data acquisition and processing detailed in this section mirror those adopted in the previous studies [13, 14]. Further details on the methodology can be found in the Additional file 1: Additional Methods.

### UL-PRx and optimal CPP assessment

UL-PRx was updated every minute and computed as a moving Pearson correlation between minute-by-minute resampled ICP and MAP data within a 30-min moving time window. The mean UL-PRx value was assessed throughout the entire monitoring duration for each patient. Our in-house algorithm underwent validation using PRx generated every minute via ICM + software [13, 14]. CPPopt, derived from UL-PRx, was computed minutely replicating the COGiTATE algorithm and using UL-PRx as input instead of PRx [24] (see Additional file 1: Additional Methods).

Every minute, we calculated the deviation between the actual CPP and CPPopt by computing the difference between CPPopt and the time-averaged CPP (ΔCPPopt). Finally, the percentage of time during which CPP deviated at least 5 mmHg above CPPopt (% above CPPopt) and the percentage of time during which CPP deviated at least 5 mmHg below CPPopt (% below CPPopt) were determined for both fatal and non-fatal cases, as well as for favorable and unfavorable patients [24, 25].

### Statistical analysis

Data are expressed as median (interquartile range, IQR), mean (standard deviation, SD), or number of patients (%). Normality of continuous variables was tested with the Shapiro–Wilks test. The mean value, time (%) spent, and the mean hourly dose above or below the defined thresholds of ICP, CPP, and UL-PRx were calculated. The mean ΔCPPopt was defined as ΔCPP = CPPopt-CPP. Demographical variables and physiological parameters were analyzed descriptively and compared between dichotomized outcome groups using the Mann–Whitney U-test for continuous variables and the Chi-square test for categorical variables.

All three models of the IMPACT score developed in adults and validated in pediatric TBI patients were used [15, 24, 25].

The age groups were designed as follows: pediatric group (0–16 y including middle adolescents [26]) and adult group [middle group (> 16 and < 70 y), older group (≥ 70 y)]. Logistic regression for each binary outcome and models were performed. The area under the receiver operating characteristic (ROC) curve (AUC) was calculated to evaluate the performance of the physiological parameters and models. The optimal threshold (best cutoff) was determined using Youden's index. The AUC (95% CI) was compared using the DeLong test. The significance was set at 0.05. The analysis was performed in the R environment.

## Results

### Study cohort

**Fig. 1:**
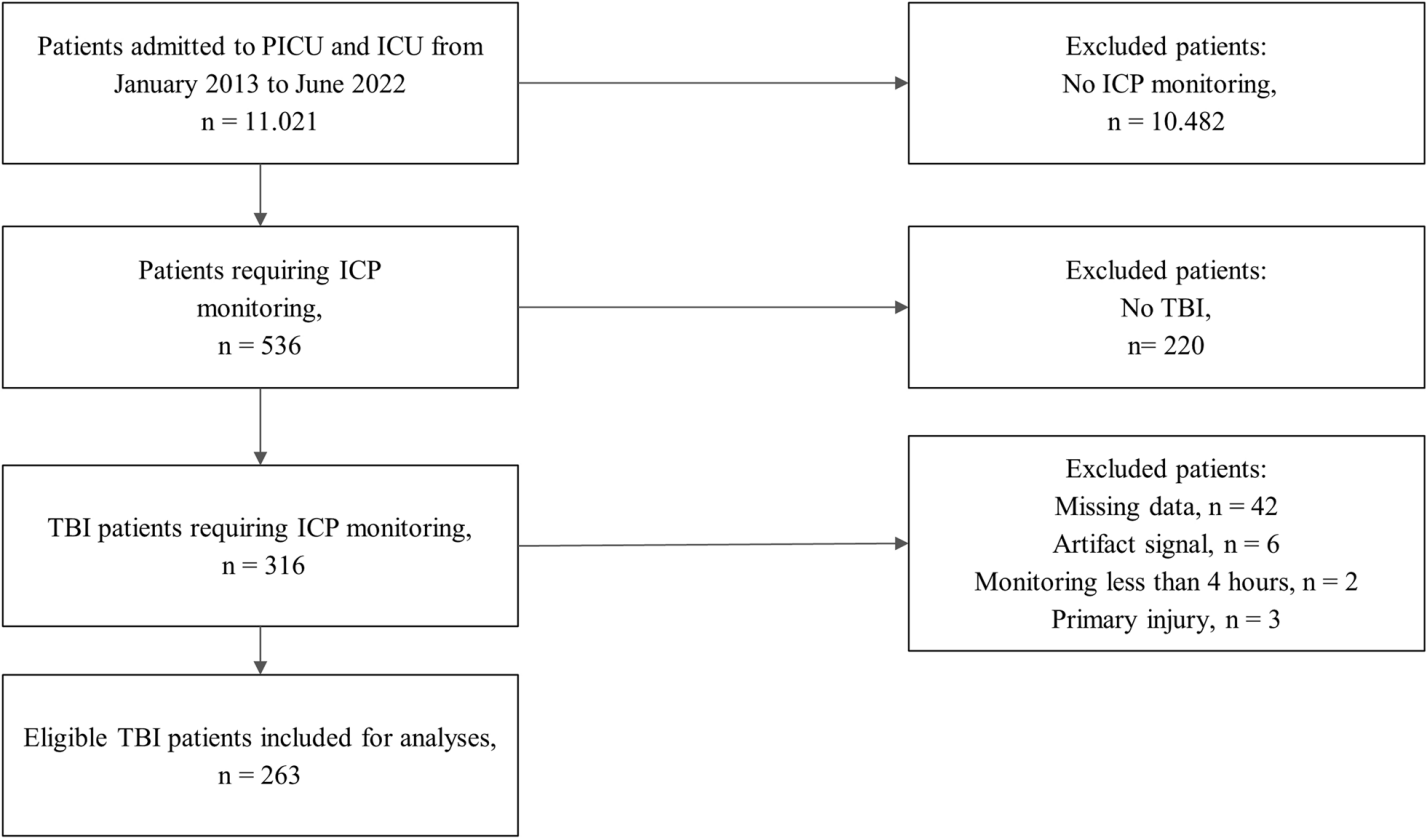
Flowchart of the study cohort. *TBI* Traumatic Brain Injury; *ICP* Intracranial Pressure; and *PICU* Pediatric Intensive Care Unit

Data on a total of 263 TBI patients admitted to the Neuro and Pediatric ICU met study criteria and were included (Fig. 1).

Median age was of 46 years (range: 2 months–85 years), with 46 patients (17.5%) ≤ 16 years. Out of the total sample, 205 patients (77.9%) were male. Most patients were victims of road accidents and had a median GCS score of 7 at admission. Demographic information, admission, and outcome clinical variables of the study cohort are summarized in Table 1.

**Table 1:** Demographic and clinical variables of the study cohort

Variables	Total
Patients, n	263
Age (years), median (IQR)	46 (23–68.50)
0–16 y, n (%)	46(17.49)
> 16 < 70 years, n (%)	159(60.46)
≥ 70 years, n (%)	58(22.05)
Sex (male), n (%)	205 (77.95)
Admission pupils response, n (%)	
Both reacting	192 (73.0)
One	18 (6.84)
None	47 (17.87)
Unknown	6 (2.28)
Admission GCS total, median (IQR)	7.0 (3.0–11.0)
Admission GCS-Motor, median (IQR)	4.0 (1.0–5.0)
Mechanism of injury, n (%)	
Road accident	126 (47.91)
Fall	112 (42.59)
Work accident	12 (4.56)
Aggression, animal aggression	10 (3.80)
Sport accident	3 (1.14)
Neurosurgical operation, n (%)^#^	182 (69.20)
Primary DC	107 (40.68)
Secondary DC	34 (12.93)
Hypoxia, n (%)	38 (14.45)
Hypotension, n (%)	41 (15.59)
Marshall CT Grade, median (IQR)	5 (3–5)
tSAH on CT, n(%)	197 (75.19)
Epidural hematoma on CT, n(%)	169 (64.26)
Glucose (mg/dL), median (IQR)	156 (128–202)
Hemoglobin (g/dL), median (IQR)	12.9 (10.70–14.50)
ICU-LOS (days), median (IQR)	19.0 (8.0–31.0)
H-LOS (days), median (IQR)	28.0 (13.0–43.0)
In-hospital mortality, n (%)	68 (25.86)
12-month mortality (Alive/Dead), n (%)*	177 (67.30)/86 (32.70)
12-month GOSE (Favorable/Unfavorable), n (%)**	105 (39.92)/ 158 (60.08)

*GCS* Glasgow Coma Score, *DC* Decompressive Craniectomy, *ICU-LOS and H-LOS* Length of Stay in Intensive Care Unit and Hospital (days), *CT* Computed Tomography, *GOSE* Glasgow Outcome Score Extended

*GOSE 2–8 versus GOSE 1, **GOSE 5–8 versus GOSE 1–4

^#^Excluding ICP catheter insertion, five patients underwent primary and secondary DC. Data are reported as n (%), proportion or median (IQR)

Neurosurgical procedure was required in 182 patients (69.2%) during hospitalization. Of these, 107 (40.7%) required primary DC within 24 h of trauma, whereas 34 patients (12.9%) underwent secondary DC (> 24 h) for intracranial hypertension refractory to maximal medical therapy. The median LOS was 28 days (13.0–43.0), the median ICU-LOS was 19 days (8.0–31.0) in the ICU. Missing values were in the following variables: GCS-Motor (n = 7) and admission pupil response (n = 6), hypoxia and hypotension (n = 3), Marshall CT score (n = 1), glucose (n = 8), and hemoglobin (n = 4).

### Outcome

Sixty-eight patients (25.9%) died before hospital discharge, while another 18 patients died within 12 months, resulting in an overall mortality rate of 86 patients (32.7%). Unfavorable outcome (GOSE 1–4) at 12 months was observed in 60.0% of patients, while favorable outcomes in 39.9% (Table 1).

### Data acquisition

A total of 2.052,763 min were extracted and 116.602 min of recording were excluded by filtering, resulting in 1.936,161 min of signal used for index processing, corresponding to approximately 1.345 consecutive days. The median monitoring time for the analyzed parameters for each patient was 5615 min (3653–9767). The median ICP of patients during their recovery in the ICU was 11.93 mmHg (8.38–16.19), with a median percentage of time with ICP > 20 mmHg of 2.9% (0.13–22.15) and a median percentage of time with ICP > 22 mmHg of 1.3% (0.03–12.72). As for CPP, the median mean value was 68.01 mmHg (61.17–73.93) with a CPP > 70 mmHg for 40.3% of the time (16.62–62.51) and a median CPPopt of 71.62 mmHg (range: 65.88–77.67). The median ΔCPPopt was 1.13 (range: -0.06–2.92). The UL-PRx median value across the studied population was 0.07 (-0.05–0.21), with a value greater than + 0.25 observed in median 38.3% of the time (31.30–47.89) and greater than + 0.30 for 36.0% of the time (29.51–45.89, Table 2).

**Table 2:** Univariate analysis for physiological parameters and outcomes

Variables	Total	Non-fatal (GOSE 2–8)	Fatal (GOSE 1)	*P* value	Favorable (GOSE 5–8)	Unfavorable (GOSE 1–4)	*P* value
Mean MAP (mmHg), median (IQR)	80 (74.59–84.97)	79.69 (74.61–84.52)	80.73 (74.59–86.56)	0.742	79.15 (74.23–83.40)	80.73 (74.88–86.22)	0.160
Mean ICP (mmHg), median (IQR)	11.93 (8.38–16.19)	11.03 (7.77–14.14)	16.75 (9.79–44.46)	**< 0.001**	11.16 (7.85–13.79)	13.24 (9.06–20.45)	**0.003**
Mean CPP (mmHg), median (IQR)	68.01 (61.17–73.93)	68.54 (64.09–74.18)	62.91 (26.89–72.32)	**< 0.001**	68.06 (63.95–73.29)	67.81 (56.71–75.10)	0.228
Mean UL-PRx, median (IQR)	0.068 (–0.046–0.211)	0.036(–0.053–0.137)	0.230 (–0.002–0.490)	**< 0.001**	0.027 (–0.049–0.128)	0.118 (–0.044–0.334)	**< 0.001**
% Time with ICP > 20 mmHg, median (IQR)	2.91 (0.13–22.15)	2.02 (0.10–10.79)	25.07 (0.32–96.20)	**< 0.001**	2.26 (0.14–9.09)	5.53 (0.14–57.67)	**0.009**
Hourly dose with ICP > 20 mmHg, median (IQR)	4.40 (0.10–46.30)	3.16 (0.03–22.65)	32.60 (0.48–1526)	**< 0.001**	4.17 (0.06–19.66)	6.95 (0.10–184.25)	**0.028**
% Time with ICP > 22 mmHg, median (IQR)	1.30 (0.03–12.72)	0.85 (0.01–6.08)	9.82 (0.18–93.95)	**< 0.001**	1.10 (0.01–5.51)	2.32 (0.04–43.26)	**0.016**
Hourly dose with ICP > 22 mmHg, median (IQR)	2.01 (0.00–28.92)	1.33 (0.00–13.38)	23.36 (0.09–)	**< 0.001**	2.01 (0.00–14.07)	2.01 (0.01–)	0.051
% Time with CPP < 60 mmHg, median (IQR)	2.60 (0.26–11.31)	1.62 (0.23–6.70)	8.34 (0.64–91.92)	**< 0.001**	2.09 (0.46–6.55)	2.74 (0.23–24.94)	0.148
% Time with CPP > 70 mmHg, median (IQR)	40.30 (16.62–62.51)	41.86 (24.34–63.81)	23.32 (0.16–57.50)	**< 0.001**	41.17 (21.91–60.88)	38.95 (6.77–64.28)	0.250
% Time with UL-PRx > 0, median (IQR)	48.01 (39.80–57.01)	46.19 (39.27–52.94)	54.21 (42.68–74.40)	**< 0.001**	47.52 (41.44–54.20)	48.65 (39.08–62.66)	0.317
% Time with UL-PRx > 0.25, median (IQR)	38.28 (31.30–47.89)	36.61 (30.71–43.52)	46.0 (33.76–67.57)	**< 0.001**	37.75 (32.47–45.21)	38.48 (30.94–54.98)	0.196
% Time with UL-PRx > 0.30, median (IQR)	36.01 (29.51–45.89)	34.69 (29.04–41.43)	43.95 (31.56–65.98)	**< 0.001**	35.85 (30.13–42.27)	36.54 (29.17–53.29)	0.193
Hourly dose of UL-PRx > 0, median (IQR)	15.93 (13.06–20.49)	15.12 (12.65–18.60)	19.59 (13.61–31.70)	**< 0.001**	15.71 (13.24–18.72)	16.16 (12.67–24.37)	0.143
Hourly dose of UL-PRx > 0.25, median (IQR)	9.39 (7.39–12.41)	8.70 (7.16–11.08)	11.98 (7.89–20.46)	**< 0.001**	9.23 (7.61–11.30)	9.48 (7.15–15.28)	0.090
Hourly dose of UL-PRx > 0.30, median (IQR)	8.26 (6.46–11.04)	7.62 (6.21–9.77)	10.63 (6.91–18.38)	**< 0.001**	8.10 (6.68–9.94)	8.40 (6.22–13.64)	0.083
Mean CPP (mmHg) opt, median (IQR)	71.62 (65.88–77.62)	71.24 (66.02–77.41)	72.80 (64.24–77.78)	0.931	70.21 (65.97–75.43)	72.31 (65.76–78.80)	0.242
Mean ΔCPPopt, median (IQR)	1.13 (− 0.06–2.92)	0.92 (–0.09–2.57)	2.13 (0.50–4.09)	**0.003**	0.77 (–0.29–2.47)	1.39 (0.24–3.32)	**0.019**
% above CPPopt, median (IQR)	24.99 (17.47–31.14)	25.66 (18.59–31.15)	23.78 (10.64–30.24)	0.060	27.0 (19.28–31.34)	24.10 (16.32–30.93)	**0.029**
% below CPPopt, median (IQR)	36.83 (29.27–43.37)	36.28 (28.51–42.22)	38.67 (31.96–53.29)	**0.017**	36.0 (27.89–41.48)	37.23 (29.97–45.94)	0.132

*MAP* Mean Arterial Pressure; *ICP* Intra-Cranial Pressure; *CPP* Cerebral Perfusion Pressure, *UL-PRx* Ultra-Low-Pressure Reactivity index*; ΔCPPopt* Δ optimal CPP; *% CPPopt plus* % of time when CPP is at least 5 mmHg lower than CPPopt; and *% CPPopt minus* % of time when CPP is at least 5 mmHg higher than CPPopt. *DC* Decompressive Craniectomy. Data are reported as median (IQR). Cerebrovascular reactivity index variables are presented as mean index value, % time above threshold, and mean hourly dose above threshold

Bold values for a *p*-value below the threshold (<0.05)

### Association with 12 months outcomes

The physiological parameters, the IMPACT covariates, and demographic and clinical characteristics of the study cohort are shown in Table 2 and Additional file 2: Table S1 and Additional file 3: Table S2, respectively, with results for both 12-month mortality and GOSE. Statistically significant differences were observed for IMPACT variables (age, GCS on admission, pupillary response, hypotension, Marshall score, and laboratories parameters) and type of trauma, with a higher incidence of TBI due to falls in both fatal and unfavorable outcome groups. Notably, there is a significant discrepancy between outcomes in patients undergoing primary DC (55.8% non-survivors vs. 33.3% survivors, respectively, *p* < 0.001, Additional file 3: Table S2) compared with patients undergoing secondary DC (3.5% non-survivors vs. 17.5% survivors). The IMPACT covariates in the cohort were largely consistent with data reported in the literature (Additional file 2: Table S1) [15, 24, 25].

The association between ICP, CPP, UL-PRx, and derived parameters, except for the mean MAP, showed a significant correlation with 12-month mortality (*p* = 0.017 to < 0.001, Table 2). Higher ICP and lower CPP values were observed in the fatal group, as well as a higher percentage of time spent with an ICP > 20 and 22 mmHg and a lower percentage of time spent with CPP < 60 mmHg. They also maintained more positive UL-PRx (% Time with UL-PRx > 0) values on average over a greater percentage of time. When comparing the favorable and unfavorable outcome groups, statistically significant results were only found for ICP, UL-PRx, and ΔCPPopt (*p* < 0.05).

### Optimal cerebral perfusion pressure

CPPopt yield was 45.6% for all TBI patients. The average CPPopt derived from UL-PRx was not statistically significant between the outcome groups. However, the mean ΔCPPopt was statistically significant in both the fatal and non-fatal groups and in the favorable and unfavorable patient groups (*p* < 0.05; Table 2). In addition, in the fatal group, the percentage of time spent with a CPP lower than 5 mmHg of CPPopt (% below CPPopt) was higher than in non-fatal group, whereas in the favorable 12 months outcome group, the percentage of time spent above 5 mmHg of CPPopt (% above CPPopt; Table 2) was higher than in the unfavorable group.

### Model's performance

ROC analysis was used to determine the diagnostic accuracy of the mean value of the physiological parameters that showed statistical significance in univariate analysis: ICP, CPP, and UL-PRx were investigated. The optimal thresholds (best cutoff) that maximized discrimination between non-survivors and survivors or between favorable and unfavorable outcome for different age groups of patients are summarized in Table 3. A good performance was found for mean UL-PRx (cutoff: 0.31), with an AUC of 0.70 (95% CI 0.62–0.78) for mortality and 0.31, with an AUC of 0.62 (95% CI 0.55–0.69) for 12-month unfavorable outcome. The cutoff of ~ 20–22 for mean ICP and of ~ 0.30 for mean UL-PRx were confirmed in different age groups, except for patients older than 70 years and patients with unfavorable outcome in middle group.

**Table 3:** Comparisons of AUC for different age groups

	12-month mortality					12-month unfavorable outcome				
	AUC (95% CI)	Optimal cutoff	Sensitivity	Specificity	*P* value	AUC (95% CI)	Optimal cutoff	Sensitivity	Specificity	*P* value
All patients 0–85 years (n = 263)										
Mean ICP	0.69 (0.61–0.77)	19.45	0.47	0.96	**< 0.001**	0.61 (0.54–0.68)	16.29	0.35	0.91	**0.003**
Mean CPP	0.64 (0.56–0.73)	57.24	0.44	0.95	0.999	0.46 (0.48–0.61)	57.62	0.27	0.95	0.886
Mean UL-PRx	0.70 (0.62–0.78)	0.31	0.47	0.98	**< 0.001**	0.62 (0.55–0.69)	0.28	0.30	0.97	**< 0.001**
Mean ΔCPPopt	0.62 (0.54–0.71)	1.46	0.61	0.63	**0.002**	0.59 (0.52–0.66)	0.91	0.61	0.54	**0.01**
Pediatric group (0–16 y, n = 46)										
Mean ICP	0.94 (0.82–1.0)	22.28	0.92	0.97	**< 0.001**	0.79 (0.65–0.93)	22.28	0.57	1.0	**< 0.001**
Mean CPP	0.90 (0.75–1.0)	38.59	0.83	1.0	0.999	0.76 (0.59–0.92)	48.93	0.57	1.0	0.999
Mean UL-PRx	0.92 (0.80–1.0)	0.33	0.83	0.97	**< 0.001**	0.74 (0.58–0.90)	0.32	0.57	1.0	**0.002**
Mean ΔCPPopt	0.65 (0.38–0.92)	0.45	1.0	0.42	0.175	0.69 (0.52–0.85)	–0.17	1.0	0.42	**0.036**
Middle group (> 16 and < 70 y, n = 159)										
Mean ICP	0.82 (0.72–0.91)	19.63	0.61	0.97	**< 0.001**	0.66 (0.58–0.74)	15.12	0.43	0.89	**< 0.001**
Mean CPP	0.78 (0.68–0.89)	59.63	0.61	0.96	0.999	0.58 (0.49–0.67)	62.18	0.33	0.92	0.959
Mean UL-PRx	0.77 (0.66–0.88)	0.29	0.61	0.97	**< 0.001**	0.62 (0.54–0.71)	0.10	0.56	0.75	**0.003**
Mean ΔCPPopt	0.65 (0.51–0.79)	1.63	0.64	0.71	**0.008**	0.54 (0.45–0.64)	4.02	0.19	0.92	0.189
Older group (≥ 70 y, n = 58)										
Mean ICP	0.62 (0.47–0.76)	13.67	0.34	0.95	0.074	0.60 (0.42–0.79)	8.21	0.67	0.71	0.188
Mean CPP	0.55 (0.39–0.70)	70.55	0.61	0.70	0.282	0.54 (0.33–0.75)	69.67	0.55	0.71	0.378
Mean UL-PRx	0.50 (0.34–0.65)	–0.05	0.26	0.90	0.483	0.47 (0.22–0.71)	0.11	0.57	0.57	0.396
Mean ΔCPPopt	0.53 (0.37–0.70)	0.85	0.36	0.80	0.665	0.53 (0.27–0.79)	0.93	0.66	0.60	0.403
Adult group (> 16 y, n = 217)										
Mean ICP	0.66 (0.58–0.75)	19.45	0.39	0.97	**< 0.001**	0.60 (0.52–0.67)	14.98	0.36	0.89	**0.010**
Mean CPP	0.63 (0.54–0.72)	59.63	0.40	0.96	0.999	0.55 (0.47–0.62)	61.0	0.25	0.95	0.867
Mean UL-PRx	0.67 (0.58–0.75)	0.31	0.40	0.99	**< 0.001**	0.61 (0.54–0.68)	0.10	0.52	0.72	**0.003**
Mean ΔCPPopt	0.62 (0.53–0.71)	2.59	0.46	0.79	**0.003**	0.57 (0.49–0.65)	2.10	0.41	0.72	**0.041**

The middle-aged and older groups constitute the adult cohort of this study (n = 217)

*ICP* Intra-Cranial Pressure; *CPP* Cerebral Perfusion Pressure; *UL-PRx* Ultra-Low-Pressure Reactivity index; *ΔCPPopt* Delta Cerebral Perfusion Pressure optimal; *AUC* Area Under Curve; *CI* Confidence Interval

Bold values for a *p*-value below the threshold (<0.05)

**Fig. 2:**
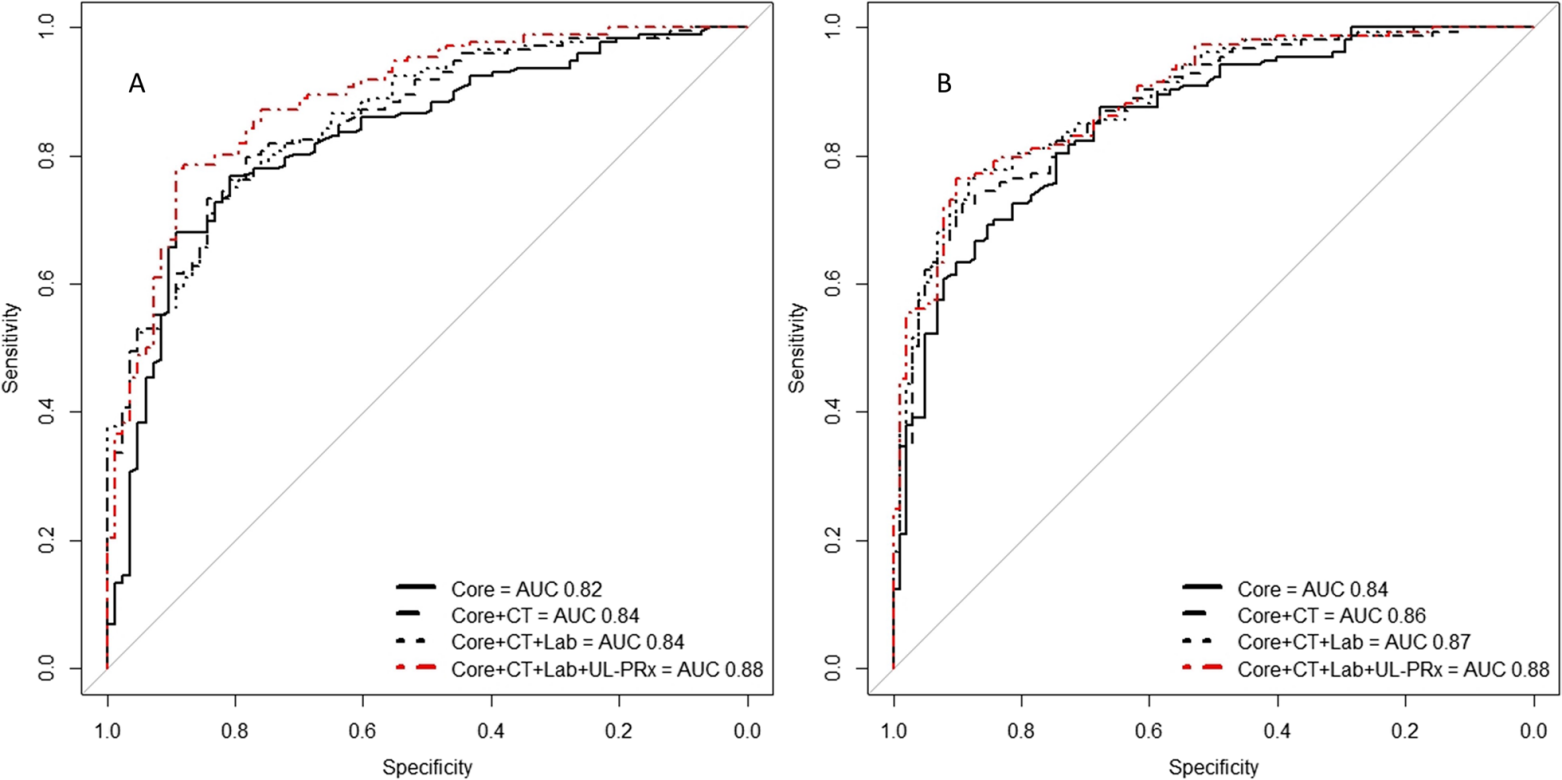
Receiver operating characteristic (ROC) curve of the predictive model of mortality (**A**) and unfavorable outcome (**B**). The figure displays ROC curves for four logistic regression models predicting mortality (**A**) and unfavorable outcome (**B**). Each model incorporates the different sets of predictor variables of the IMPACT models (core, core + CT, core + CT + lab). The last model further includes the mean UL-PRx. The legend indicates the corresponding AUC values for each model. The IMPACT core + CT + lab + UL-PRx model demonstrated significantly superior performance for predicting both mortality and unfavorable outcomes

To evaluate the discriminatory performance of UL-PRx when adjusted for the IMPACT models, the AUCs of the ROC curves were compared (Additional file 4: Table S3). After adjustment for the IMPACT Core + CT + Lab parameters, mean UL-PRx remained significantly associated with 12-month mortality and unfavorable outcome (Fig. 2 A and B). The IMPACT core + CT + Lab + UL-PRx model showed significantly better performance for both mortality and unfavorable outcome (AUC 0.88; 95% CI 0.84–0.93 and AUC 0.88; 95% CI 0.84–0.92, respectively; Additional file 4: Table S3), with UL-PRx significantly associated with the outcome measures (Additional file 5: Table S4). UL-PRx remained statistically significant in the logistic model that considers IMPACT core + CT + Lab + UL-PRx, even when adjusted for hemicraniectomy (OR (95% CI) for 12-month mortality: 8.04, 1.26–51.08, *p* = 0.027; 12-month unfavorable outcome: 7.66, 1.21–48.32, *p* = 0.030; data not shown).

These results were confirmed when younger and older groups were excluded by the analysis, resulting in a sample of 159 patients ranging from > 16 to < 70 years. However, the selection of a more homogeneous groups of patients ameliorates the fit of the models (Additional file 6: Table S5).

The DeLong test showed statistical significance when comparing the IMPACT core model with IMPACT core + CT + Lab, and UL-PRx for of 12-month mortality and unfavorable outcome (*p* < 0.01). Subgroup analysis of the pediatric patient cohort (ages 0–16) revealed that the age variable within the IMPACT core model had no significant effect on the performance of the pediatric core model (*p* = 0.61, data not shown).

**Fig. 3:**
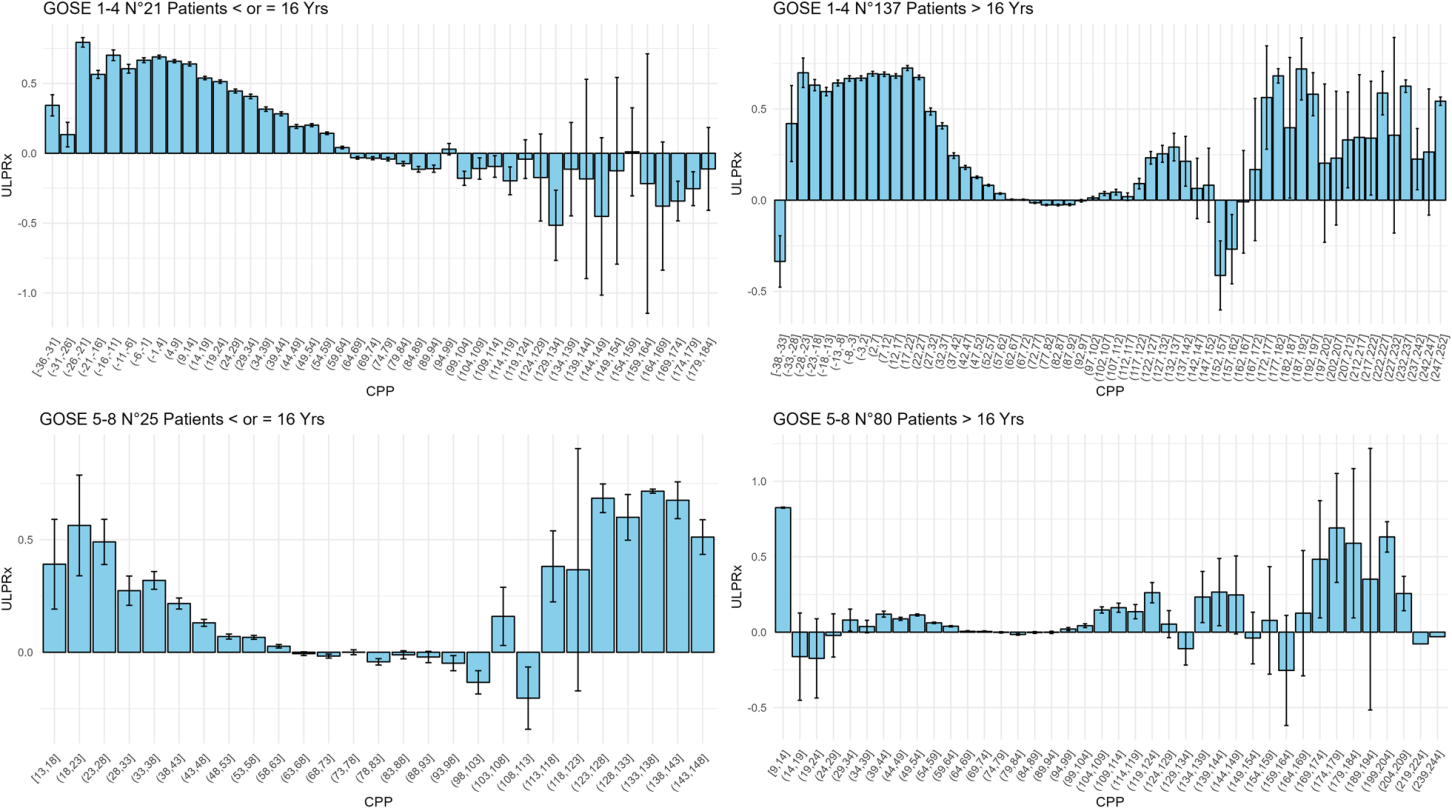
A–D Relationship between CPP and UL-PRx, stratified by age and GOSE. The combined plot illustrates a comparative analysis of ultra-low-pressure reactivity index (UL-PRx) dynamics in patients with traumatic brain injury, specifically examining the relationship between cerebral perfusion pressure (CPP) and UL-PRx. The plot is divided into four quadrants, each focusing on different patient groups based on Glasgow Outcome Scale Extended (GOSE) scores and age categories. The upper left quadrant examines autoregulation patterns for GOSE 1–4 patients aged 16 years or younger, while the upper right quadrant examines patients older than 16 years. The lower left quadrant provides insights into UL-PRx dynamics for GOSE 5–8 patients aged 16 years or younger, while the lower right quadrant examines the same for those aged 16 years or older. Each bar represents the mean UL-PRx value for specific CPP groups, with error bars indicating 95% confidence intervals. In the top panels, patients with poorer GOSE scores show a trend toward higher UL-PRx values, both in the pediatric and adult populations. Conversely, the lower panels, representing patients with higher CPP and positive results, show a trend toward values close to or predominantly negative UL-PRx

**Fig. 4:**
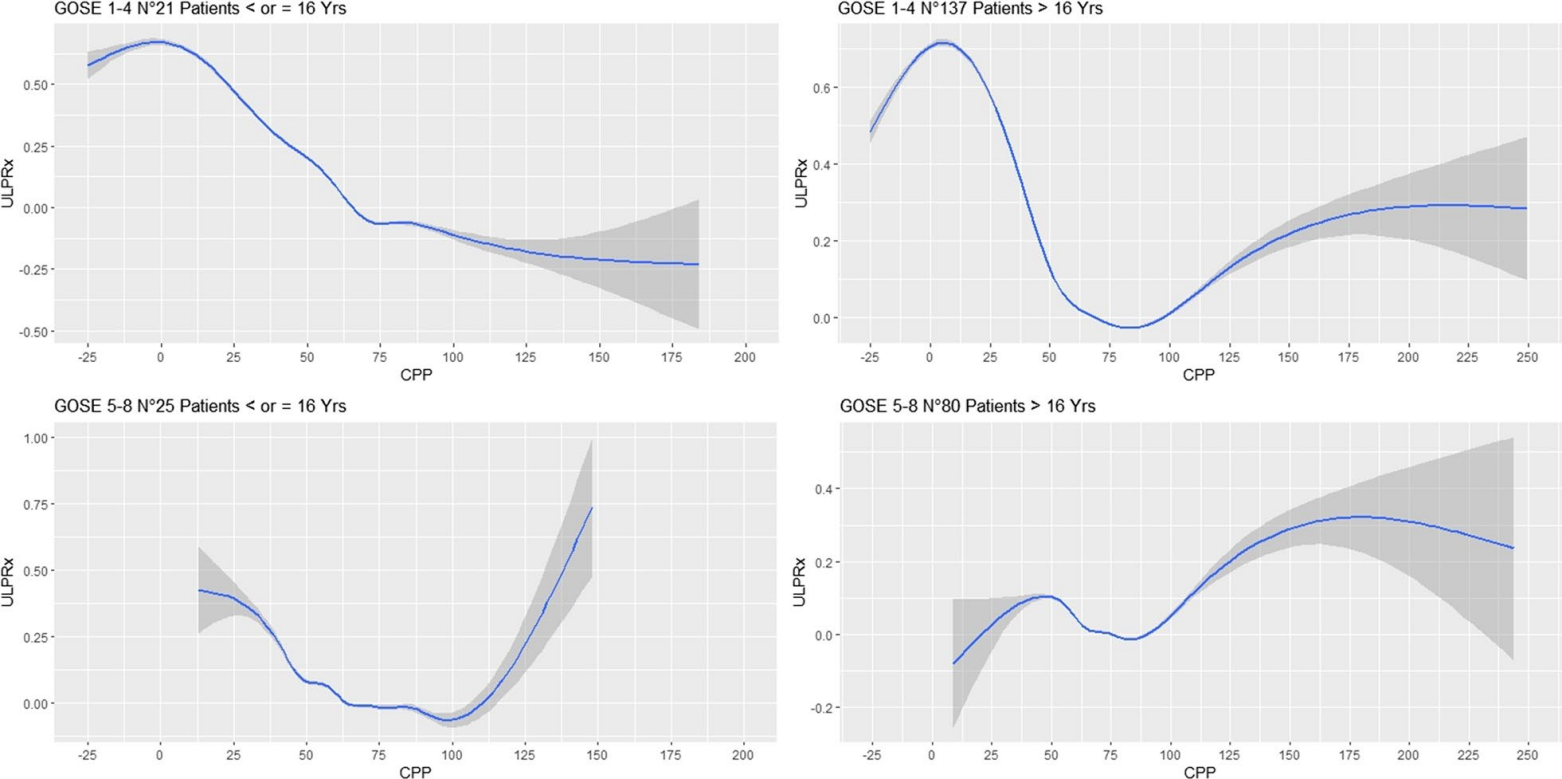
**A**–**D** Visual examination of UL-PRx dynamics and CPP stratified by age and GOSE. The results, visually represented in the figure by smoothing curves and confidence intervals, clearly show a gradual escalation of high UL-PRx values indicating impaired cerebral autoregulation (CA), especially at low CPP values (CPP below the threshold of about 50 mmHg). Of note, individuals with unfavorable outcomes tend to have lower CPP values and thus show a more pronounced increase in UL-PRx. The figure provides valuable insight into the relationship between CPP and UL-PRx in different age groups and categories of the Glasgow Outcome Scale and sheds light on the potential impact on cerebral autoregulation in patients with traumatic brain injury

In both pediatric and adult patients, the visualization of UL-PRx acquisition plotted against CPP values showed an increase toward the upper and positive limits of UL-PRx values at lower CPP in patients with unfavorable outcomes (Figs. 3A–D and 4A–D and Additional file 7: Figure S1).

## Discussion

The main finding of this study confirms that UL-PRx, derived from data sampled at a very low rate and adjusted for all IMPACT models, may be considered as a predictor associated with 12-month mortality and unfavorable outcomes in a cohort of pediatric and adult patients with TBI. In addition, the ΔCPPopt, calculated as the difference between CPPopt derived by UL-PRx and CPP, showed significant differences in outcomes in patients with fatal and unfavorable prognosis.

This study extends and complements two previous studies investigating the use of data sampled at ultra-low frequencies in adult (n = 164) and pediatric (n = 47) patients with TBI [13, 14]. It goes beyond the mere pooling of two previous analyses. Both cohorts reflect real-life scenarios and have direct clinical relevance considering that trauma facilities often treat patients of different ages. The combined dataset allows for subgroup analyses that reveal potential age-specific nuances in UL-PRx performance. It reconfirms that it is possible to extract valuable information from data acquired at an approximate 5-min interval. When the three IMPACT models (Core, Core + CT, and Core + CT + Lab) were applied to the cohort, their performance showed a progressive increase in AUC, indicating a good fit of the data. Inclusion of UL-PRx in the final model resulted in the highest performance.

Few studies in the literature have investigated the integration of PRx or derived indices in prognostic models [27, 28]. In a comparison between L-PRx and PRx, L-PRx indices were less effective in predicting outcomes compared to PRx [29], with AUC values of approximately 0.63 for fatal outcomes and 0.54 for unfavorable outcomes [28]. Interestingly, the UL-PRx showed different discrimination properties in different age groups. It showed optimal discriminatory ability in the pediatric cohort and retained strong predictive ability in middle group, in contrast with the previous results with L-PRx data [28]. In the population aged over 70 years, UL-PRx, along with ICP and CPP values, loses predictive power. This observation suggests that the elderly may exhibit different physiological patterns that are influenced by age- and frailty-related factors [20, 29]. Moreover, it is conceivable that UL-PRx is more evident in contexts characterized by substantial fluctuations in ICP and CPP, and its predictive efficacy appears to be influenced, particularly in the younger age group and in adults, where thresholds for ICP and CPP have a significant impact. ICP thresholds in adult patients reveal a cutoff value of 20 mmHg. In contrast, in pediatric patients, there is evidence of an optimal ICP threshold of 22.5 mmHg, which is higher than the 20 mmHg established in the guidelines [23]. It is important to note that we used the Youden index rather than the sequential Chi-square method for the analysis [7]. However, the fact that the pediatric group maintained higher levels of ICP compared to adults could be due to several factors. The thin skullcap of children may make monitoring intracranial pressure more difficult, leading neurosurgeons to be initially reluctant to use bolt systems or to measure ICP in very young patients and to postpone catheter insertion until more critical conditions occur [30]. In addition, a greater propensity to prolong treatment and avoid treatment discontinuation in pediatric cases may contribute to extending ICP monitoring over a longer period. While open sutures and fontanelles provide some buffering capacity for ICP, especially when intracranial volume increases gradually, TBI in young patients can lead to rapid escalation of intracranial volume. The smaller size and limited capacity of a child's skull mean that increased compliance may be quickly exhausted [31]. Any increase in brain tissue, cerebrospinal fluid, or blood inside the skull can lead to a greater increase in intracranial pressure. However, the similar outcomes observed in pediatric patients compared to adults, suggest that the compensatory mechanisms for intracranial hypertension are better tolerated in pediatric cases.

Another noteworthy observation is that approximately 70% of patients underwent neurosurgical procedures, with more than half undergoing primary or secondary DC. Nevertheless, UL-PRx maintained a strong association with the outcome, implying that it could be reliable as a predictive value even in scenarios involving severe TBI patients undergoing intensive neurosurgical treatment.

On average, CPPopt could not be calculated for more than half of the monitoring time. We do not rule out that the algorithm derived from COGiTATE which was originally designed to process high-frequency input data, needs to be modified to account for the low-frequency nature of the data, and that a customized algorithm for calculating CPP with UL-PRx could be developed in the future. Calculation of CPPopt from UL-PRx using the algorithm proposed in the COGiTATE protocol yields a ΔCPPopt that exhibits a significant association with patient outcomes. This correlation is observed in both fatal and non-fatal cases, as well as in patients categorized as favorable or unfavorable [30]. More interestingly, among patients with fatal outcomes, those who spent a lower percentage of time below the CPPopt threshold and among favorable patients, those who exceeded the CPPopt threshold for a higher percentage of time had better clinical outcomes. These findings suggest the possible identification of a critical zone where the risk of worse outcome may be detectable. Remarkably, these findings are from ultra-low-frequency sampling data, which may offer sufficient resolution to provide preliminary insights into the state of CA. However, we cannot exclude that the algorithm derived from COGiTATE needs to be adapted to our frequency data [30]. Indeed, the low frequency and the results visually represented by the smoothing curve in Fig. 4 make it clear that worse outcomes are associated with a curve that diverges toward positive UL-PRx values (reduced autoregulation), particularly in the context of negative cerebral perfusion pressure. These observations may be related to the findings of Beqiri, who recently demonstrated that a CPP below the lower limit of reactivity (LLR) during the first 7 days post injury positively correlates with 6-month mortality, supporting future investigations on personalized and dynamic CPP targets in the treatment of TBI [31]. On the contrary, the consistently positive values of the smoothing curve in patients with favorable outcome suggest that maintaining higher perfusion pressure values is not associated with worsening conditions.

A system capable of extracting information about autoregulation at a sampling frequency more than 100 times lower than that of PRx could make this monitoring capability accessible to many ICUs [8]. A recent study by Zoerle [32] highlighted that within the Center TBI consortium, only a quarter (approximately 21 of 80) had a continuous monitoring system for ICP and CPP, while others reported data hourly or every 2 h. Hence, examining low sampling rate data to identify potential correlations with autoregulation proves useful, especially in centers where automatic high sampling rates are not available [8, 33]. Knowledge of the UL-PRx could be of practical use for several purposes, primarily as a type of continuous passive pressure test to apply a different approach to CPP or ICP treatment. Howells demonstrated that 'pressure-active' TBI patients, i.e., patients with preserved autoregulation, benefited from CPP-targeted therapy, whereas 'pressure-passive' patients benefited from ICP-targeted therapy [33]. In this perspective, UL-PRx could be used to similarly guide these two different approaches. More recently, Wettervik has demonstrated that PRx can be employed as an alternative method to assess an anticipatory and safe CPP range when CPPopt is not available [34]. Because CPPopt often cannot be calculated, targeting CPP to the absolute PRx value resulted as another option. A wider cerebral perfusion pressure range from 55 to 75 mm Hg can be tolerated if cerebral pressure autoregulation is intact and the pressure reactivity index is below zero, whereas this range can be much narrower, especially for the upper cerebral perfusion pressure threshold at a higher pressure reactivity index above zero [34].

### Limits and hypothesis

This study has several limitations. The study encompasses a population of TBI patients with a wide age range (from a few months to 85 years), which could be considered a limitation because of the heterogeneity of the cohort. In our study, patients who had undergone neurosurgical operations and DC were not excluded. However, the fact that UL-PRx showed an association with outcome suggests that may also be a reliable marker of CA in craniectomized patients [7]. Another important limitation in CPPopt evaluation is the lower percentage of time it could be detected. In this case, in less than 50% of the time (45.6%), although the UL-PRx has good discrimination ability, it is important to note that the effectiveness of the method may be limited by the ultra-low sampling frequency, which restricts the representation to frequencies below 0.0017 Hz. This limitation is noteworthy in that research suggests that the optimal discrimination frequency for distinguishing between intact and impaired cerebral autoregulation is approximately 0.017 Hz [35]. No comparative analysis of the performance of PRx and UL-PRx for outcome prediction was performed, as the algorithms (working with high- and low-resolution ABP and ICP data) perform differently. Additionally, an arbitrary age limit for pediatric patients was set up to the age of 16. This decision was guided by certain considerations. According to the American Academy of Pediatrics [26], 15–17 years of age is considered the middle pediatric age group, so we set the group at the midpoint (16 years). From a physiological point of view, patients aged ≤ 16 years are different from adults [36, 37]. This choice is in line with some other papers on traumatology [38, 39].

## Conclusions

In summary, this study highlights the significance of UL-PRx as a tool for the evaluation of CA and as a valuable predictor for patients with TBI. The findings highlight the potential clinical utility of UL-PRx and its adaptability across different age groups, even after adjustment for IMPACT models. Furthermore, the correlation between UL-PRx and CPPopt with outcome suggests the potential for more targeted CPP management in TBI patients. Despite some limitations, this study paves the way for further research and clinical applications promoting the potential enhancement of TBI management.

### Supplementary Information

**Additional file 1.** Additional Methods.

**Additional file 2. Table S1.** Univariate analysis for IMPACT variables and outcomes.

**Additional file 3. Table S2.** Demographic and clinical characteristics of the study cohort by outcome.

**Additional file 4. Table S3.** Multivariate logistic regression analysis.

**Additional file 5. Table S4.** Multivariable Logistic Regression Analysis at 12 months.

**Additional file 6. Table S5.** Multivariate logistic regression analysis in middle groups

**Additional file 7.** Figure S1.

## Data Availability

The datasets used and/or analyzed during this study are not publicly available but are available from the corresponding authors on reasonable request.
