# Supplementary material for: Cerebral autoregulation in traumatic brain injury: ultra-low-frequency pressure reactivity index and intracranial pressure across age groups
Source: Crit Care. 2024 Jan 23;28:33. doi: 10.1186/s13054-024-04814-5 (PMC10807228; doi:10.1186/s13054-024-04814-5)
Supplement: Supplementary file 1 — Additional file 1. Additional Methods. [file 13054_2024_4814_MOESM1_ESM.docx]

**ADDITIONAL METHODS**

The following section provides an overview of how the data of the ICP-derived indices were obtained, elaborated and managed, integrating the methods described in the manuscript. All the procedures described below precede the integration of the data into a statistical analysis together with the clinical data.

**Road map of data extraction, storage and elaboration**

- Query data (physiological measures: MAP, ICP, CPP) from hospital database system GE’s Centricity Critical Care, Chalfont St. Giles, UK) at ~0.0033 Hz (about an average period of 5 minutes) through interactive SQL (Toad ® for SQL, Quest Software, California).
- Storage of the data extracted from the database as .csv files.
- Manual data filtering and spikes identification of time series*
- Load filtered .csv files in Matlab
- Automatic discarding of periods with incomplete recordings lasting more than 25 minutes and time series shorter than 60 minutes
- Time series synchronization and minute-by-minute resampling
- Minutely calculation of UL-PRx using ICP and ABP resampled data
- Minutely calculation of CPPopt using resampled CPP and UL-PRx data
- Store .mat file containing ICP, MAP, CPP, UL-PRx and CPPopt data minute-by-minute
- Load .mat files in Matlab and calculation of physiological indices:
  - Mean MAP
  - Mean ICP
  - Mean CPP
  - % Time with ICP >20 mmHg
  - % Time with ICP >22 mmHg
  - Hourly dose with ICP >20 mmHg
  - Hourly dose with ICP >22 mmHg
  - % Time with CPP <60 mmHg
  - % Time with CPP <70 mmHg
  - % Time with UL-PRx > 0
  - % Time with UL-PRx > 0.25
  - % Time with UL-PRx > 0.30
  - Mean CPPopt
  - Mean ΔCPP opt
  - % above CPPopt
  - % below CPPopt

*Artifacts handling

To ensure the accuracy of the data with respect to various artifacts, the time series were subjected to a double-check involving an intensivist and a biomedical engineer trained by medical staff and blended from clinical data. During this process, if a parameter value matched the maximum measurable value of the sensor (full scale), that data point was excluded from the analysis. If an abnormal value showed a significant change while other vital signs (heart rate, temperature, MAP and ICP) acquired at the same time points remained constant, it was considered an artifact and removed. In case of uncertainty, the medical records were searched for symptoms or situations that could explain abnormally high or low values (double check by two intensivists).

**UL-PRx calculation**

UL-PRx was calculated every minute by performing a Pearson correlation between the minute-to-minute linearly interpolated values of ICP and MAP within a 30-minute mobile temporal window, comprising 15 minutes before and 14 minutes after the minute under consideration.

**Procedures for CPPopt calculation**

The following procedures for algorithm development reflect the method implemented in the COGiTATE protocol [1], which is a further development of the approach previously described in the appendix of the article by Aries et al [2].

The CPPopt values were calculated every minute in different time windows between 2 and 8 hours with an increment of 10 minutes. The CPPopt values of the time windows were combined with a weighted average. This average was determined by the product of CPPopt and the value R^2. R^2 was set to zero if the fitted curve is not U-shaped.

The resulting CPPopt time series was subjected to an exponentially weighted average filter of 2 hours duration, calculated as (1-0,1)^k, where k is the distance in number of samples from the current sample.

The procedure for calculating CPPopt of time windows was performed as follows:

1. Preprocessing:

- Apply a 5-minute median filter to the CPP time series.
- Fisher transformation of UL-PRx.

1. Treatment of missing data:

- Ensure that the percentage of missing data does not exceed 50%; otherwise, an invalid CPPopt value is returned.

1. Data binning:

- Bind the data by CPP values and divide into 5-mm Hg sections (bin) from 40 mmHg to 120 mmHg on a UL-PRx vs. CPP error bar.
- Discard the first and last CPP bin.
- Each CPP bin must account for at least 3% of the total amount of data; otherwise, an invalid CPPopt value is returned.

1. CPPbest identification:

- Identify CPPbest as the CPP bin with the minimum mean UL-PRx value.

1. Curve fitting:

- Fit a second order polynomial representing a parabola to the mean UL-PRx vs. CPP error bars.
- Criteria for curve fitting:
  - The mean UL-PRx values in the final two bins at both ends of the curve should follow the appropriate sequence based on the section of the convex U-shaped curve. If the edge bins fail to meet this criterion, they are omitted, and the fitting procedure is repeated.
  - The data used for the fit must account for 50% of all data points within the analyzed time window.
  - The values used in the fitting process must represent at least 50% of the PRx range within that window.
  - The number of bins must be at least 4.
  - The PRx range must be at least 0.2 and limited to a value between -0.3 and 0.6.
  - The fitted curve must contain CPPbest.
  - The coefficient of determination R^2 must be at least 0.2 for the fitted curve, also calculated for excluded bins. R^2 is set to zero if the curve does not follow the convex shape.
- If the criteria are not met, an invalid CPPopt value is returned.

1. CPPopt determination:

- CPPopt for the time window is the CPP value at which the fitted curve reaches the smallest UL-PRx value.

**REFERENCES**

1. Beqiri E, Smielewski P, Robba C, Czosnyka M, Cabeleira MT, Tas J, Donnelly J, Outtrim JG, Hutchinson P, Menon D, Meyfroidt G, Depreitere B, Aries MJ, Ercole A. Feasibility of individualised severe traumatic brain injury management using an automated assessment of optimal cerebral perfusion pressure: the COGiTATE phase II study protocol. BMJ Open. 2019 Sep 20;9(9):e030727. doi: 10.1136/bmjopen-2019-030727. PMID: 31542757; PMCID: PMC6756360.
2. Aries MJ, Czosnyka M, Budohoski KP, Steiner LA, Lavinio A, Kolias AG, Hutchinson PJ, Brady KM, Menon DK, Pickard JD, Smielewski P. Continuous determination of optimal cerebral perfusion pressure in traumatic brain injury. Crit Care Med. 2012 Aug;40(8):2456-63. doi: 10.1097/CCM.0b013e3182514eb6. PMID: 22622398.
