# Supplementary material for: Cerebral autoregulation in traumatic brain injury: ultra-low-frequency pressure reactivity index and intracranial pressure across age groups
Source: Crit Care. 2024 Jan 23;28:33. doi: 10.1186/s13054-024-04814-5 (PMC10807228; doi:10.1186/s13054-024-04814-5)
Supplement: Supplementary file 2 — Additional file 2. Table S1. Univariate analysis for IMPACT variables and outcomes. [file 13054_2024_4814_MOESM2_ESM.docx]

**TABLE S1. Univariate analysis for IMPACT variables and outcomes**

| **Variables** | **Non-fatal (GOSE 2-8)** | **Fatal (GOSE 1)** | ***p-value*** | **Favorable (GOSE 5-8)** | **Unfavorable (GOSE 1-4)** | ***p-value*** |
| --- | --- | --- | --- | --- | --- | --- |
| **IMPACT- CORE variables** | | | | | | |
| Age (yrs), median (IQR) | 37.0 (20.0-56) | 68.50 (44.75-74.0) | **< 0.001** | 32.0 (16.40-52.0) | 60.5 (34.0-71.75) | **< 0.001** |
| **Admission pupil response, n (%)** |  |  |  |  |  |  |
| Both reacting | 145 (81.92) | 47 (54.65) | ref | 97 (92.38) | 95 (60.13) | ref |
| One | 13 (7.34) | 5 (5.81) | 0.760 | 4 (3.81) | 14 (8.86) | **0.029** |
| None | 17 (9.60) | 30 (34.88) | **< 0.001** | 3 (2.86) | 44 (27.85) | <**0.001** |
| Unknown | 2 (1.13) | 4 (4.65) | **-** | 1 (0.95) | 5 (3.16) | 0.140 |
| Admission GCS-motor, median (IQR) | 4.0(2.0-5.0) | 3.0(1.0-5.0) | **0.04** | 4.0(2.5-6.0) | 3.0(1.0-5.0) | **0.004** |
| **IMPACT- CT variables** | | | | | | |
| Hypoxia, n (%) | 23 (13.07) | 15 (17.86) | 0.404 | 14 (13.46) | 24 (15.38) | 0.810 |
| Hypotension, n (%) | 21 (11.93) | 20 (23.81) | **0.022** | 10 (9.62) | 31 (19.87) | **< 0.040** |
| Marshall CT Grade, median (IQR) | 5.0(2.0-5.0) | 5.0(5.0-5.0) | **< 0.001** | 5.0(2.0-5.0) | 5.0(5.0-5.0) | **< 0.001** |
| **Marshall CT Classification, n (%)** |  |  |  |  |  |  |
| diffuse injury I | 6 (3.39) | 1 (1.18) | 0.194 | 5 (4.76) | 2 (1.27) | 0.043 |
| diffuse injury II | 40 (22.60) | 6 (7.06) | **0.001** | 30 (28.57) | 16 (10.19) | **<0.001** |
| diffuse injury III or IV | 23 (12.99) | 4 (4.71) | **0.015** | 14 (13.33) | 13 (8.28) | **0.034** |
| V or VI^#^ | 108 (61.02) | 74 (87.06) | ref | 56 (53.33) | 126 (80.25) | ref |
| tSAH on CT, n(%) | 129 (72.88) | 68 (80.0) | 0.273 | 67 (63.81) | 130 (82.80) | **< 0.001** |
| Epidural hematoma on CT, n(%) | 68 (38.42) | 25(29.41) | 0.197 | 36 (34.29) | 57 (36.31) | 0.839 |
| **IMPACT- LAB variables** | | | | | | |
| Glucose (mg/dL), median (IQR) | 150.0 (124.0-184.0) | 172.0 (136.0-231.0) | **0.005** | 142.0 (114.25-165.0) | 174.0 (137.0-218.0) | **< 0.001** |
| Haemoglobin (g/dL), median (IQR) | 13.30 (11.30-14.65) | 12.65 (10.28-14.0) | **0.019** | 13.80 (11.50-14.93) | 12.80 (10.45-14.20) | **0.005** |

GCS = Glasgow Coma Score; CT = computed tomography; tSAH = traumatic Sub-Arachnoid Hemorrhage; GOSE = Glasgow Outcome Score. Data are reported as n (%) or median (IQR). ref= reference category. ^#^evacuated or non-evacuated mass lesion.
