# Supplementary material for: Cerebral autoregulation in traumatic brain injury: ultra-low-frequency pressure reactivity index and intracranial pressure across age groups
Source: Crit Care. 2024 Jan 23;28:33. doi: 10.1186/s13054-024-04814-5 (PMC10807228; doi:10.1186/s13054-024-04814-5)
Supplement: Supplementary file 3 — Additional file 3. Table S2. Demographic and clinical characteristics of the study cohort by outcome. [file 13054_2024_4814_MOESM3_ESM.docx]

**TABLE S2. Demographic and clinical characteristics of the study cohort by outcome**

| **Variables** | **Non-fatal (GOSE 2-8)** | **Fatal (GOSE 1)** | ***p-value*** | **Favorable (GOSE 5-8)** | **Unfavorable (GOSE 1-4)** | ***p-value*** |
| --- | --- | --- | --- | --- | --- | --- |
| Number of patients | 177 (67.30) | 86 (32.70) |  | 105 (39.92) | 158 (60.08) |  |
| Sex (male), n (%) | 133 (75.14) | 72 (83.72) | 0.157 | 123 (77.85) | 82 (78.10) | 1.0 |
| Admission GCS, median (IQR) | 7.0 (4.0-12.0) | 5.0 (3.0-10.0) | **0.030** | 7.0 (5.0-13.0) | 6.0 (3.0-10.0) | **< 0.05** |
| **Mechanism of injury, n (%)** |  |  |  |  |  |  |
| Road accident | 93 (52.54) | 33 (38.37) | ref | 63 (60.0) | 63 (39.87) | ref |
| Fall | 63 (35.59) | 49 (56.98) | **0.005** | 27 (25.71) | 85 (53.80) | **< 0.001** |
| Work accident | 10 (5.65) | 2 (2.33) | 0.474 | 6 (5.71) | 6 (3.80) | 1.0 |
| Aggression, animal aggression | 9 (5.08) | 1 (1.16) | 0.280 | 7 (6.67) | 3 (1.90) | 0.234 |
| Sport accident | 2 (1.13) | 1 (1.16) | 0.782 | 2 (1.90) | 1 (0.63) | 0.575 |
| Neurosurgical operation  (except ICP catheter insertion), n (%) | 116 (65.54) | 66 (76.74) | 0.064 | 62 (59.05) | 120 (75.95) | **< 0.05** |
| Primary DC | 59 (33.33) | 48 (55.81) | **< 0.001** | 26 (24.76) | 81 (51.27) | **< 0.001** |
| Secondary DC | 31 (17.51) | 3 (3.49) | **0.003** | 15 (14.29) | 19 (12.03) | 0.728 |
| ICU-LOS (days), median (IQR) | 23.0 (15.0-33.0) | 7.0 (3.0-17.50) | **< 0.001** | 19.0 (12.0-27.0) | 19.0 (6.0-33.0) | 0.793 |
| H-LOS (days), median (IQR) | 34.0 (24.0-46.0) | 7.0 (3.0-20.25) | **< 0.001** | 28.0 (22.0-40.0) | 25.50 (6.0-46.0) | 0.156 |

GCS = Glasgow Coma Score; DC= Decompressive Craniectomy; ICU-LOS and H-LOS= Length of Stay in Intensive Care Unit and Hospital (days). Data are reported as n (%), proportion or median (IQR). ref= reference category
