# Supplementary material for: Cerebral autoregulation in traumatic brain injury: ultra-low-frequency pressure reactivity index and intracranial pressure across age groups
Source: Crit Care. 2024 Jan 23;28:33. doi: 10.1186/s13054-024-04814-5 (PMC10807228; doi:10.1186/s13054-024-04814-5)
Supplement: Supplementary file 4 — Additional file 4. Table S3. Multivariate logistic regression analysis. [file 13054_2024_4814_MOESM4_ESM.docx]

**Table S3. Multivariate logistic regression analysis**

| **12-month mortality** | | | |
| --- | --- | --- | --- |
| **Model description** | **AUC (95% CI)** | **AIC** | **Adjusted R^2^** |
| **IMPACT- Core** | 0.82 (0.77-0.88) | 262.28 | 0.29 |
| **IMPACT- Core + CT** | 0.84 (0.79-0.89) | 254.67 | 0.31 |
| **IMPACT- Core + CT + Lab** | 0.84 (0.79-0.89) | 255.72 | 0.30 |
| **IMPACT- Core + CT + Mean UL-PRx** | 0.88 (0.84-0.93) | 229.66 | 0.38 |
| **IMPACT- Core + CT + Lab + Mean UL-PRx** | 0.88 (0.84-0.93) | 230.69 | 0.37 |
| **12-month unfavourable outcome** | | | |
| **Model description** | **AUC (95% CI)** | **AIC** | **Adjusted R^2^** |
| **IMPACT- Core** | 0.84 (0.80-0.89) | 261.76 | 0.28 |
| **IMPACT- Core + CT** | 0.86 (0.82-0.91) | 260.39 | 0.32 |
| **IMPACT- Core + CT + Lab** | 0.87 (0.83-0.92) | 246.60 | 0.36 |
| **IMPACT- Core + CT + Mean UL-PRx** | 0.87 (0.82-0.91) | 256.48 | 0.34 |
| **IMPACT- Core + CT + Lab + Mean UL-PRx** | 0.88 (0.84-0.92) | 243.31 | 0.37 |

AUC= Area Under the Curve. CI = Confidence Interval. AIC= Akaike Information Criterion. p value < 0.001 for all the models.
