# Supplementary material for: Cerebral autoregulation in traumatic brain injury: ultra-low-frequency pressure reactivity index and intracranial pressure across age groups
Source: Crit Care. 2024 Jan 23;28:33. doi: 10.1186/s13054-024-04814-5 (PMC10807228; doi:10.1186/s13054-024-04814-5)
Supplement: Supplementary file 5 — Additional file 5. Table S4. Multivariable Logistic Regression Analysis at 12 months. [file 13054_2024_4814_MOESM5_ESM.docx]

**Table S4. Multivariable Logistic Regression Analysis at 12 months**

| **Model** | **12-month mortality** | | | **12-month unfavorable outcome** | | | |
| --- | --- | --- | --- | --- | --- | --- | --- |
|  | **OR (95% CI)** | | ***p-value*** | | **OR (95% CI)** | | ***p-value*** |
| **IMPACT- Core + CT + Lab + Mean UL-PRx** | | | | | | | |
| Age | 1.06 (1.04-1.08) | **<0.001** | | 1.05 (1.03-1.07) | | **<0.001** | |
| Admission pupil response |  |  | |  | |  | |
| *Both reacting* | ref | **-** | |  | | **-** | |
| *One* | 1.73 (0.40-7.41) | ns | | 5.62 (1.37-23.08) | | **0.016** | |
| *None* | 4.09 (1.43-11.70) | **0.009** | | 1.58 (0.36-6.90)* | | **<0.001** | |
| *Unknown* | 4.47 (0.-) | ns | | 1.98 (0.-) | | ns | |
| Admission GCS-motor | 0.86 (0.67-1.09) | ns | | 0.73 (0.61- 0.98) | | **0.030** | |
| Hypoxia | 1.12 (0.29-4.31) | ns | | 2.41 (0.61-9.54) | | ns | |
| Hypotension | 0.38 (0.11-1.41) | ns | | 0.51 (0.14-1.90) | | ns | |
| Marshall CT Grade |  |  | |  | |  | |
| *diffuse injury I* | 0.38 (0.02-5.76) | ns | | 0.34 (0.04-3.23) | | ns | |
| *diffuse injury II* | 0.98 (0.32-2.96) | ns | | 0.56 (0.23-1.41) | | ns | |
| *diffuse injury III or IV* | 0.35 (0.07- 1.70) | ns | | 0.53 (0.16- 1.71) | | ns | |
| *V or VI* | ref | **-** | | ref | | **-** | |
| tSAH on CT | 0.52 (0.24-1.13) | ns | | 1.45 (0.58-3.62) | | ns | |
| Epidural hematoma on CT | 0.57 (0.21- 1.58) | ns | | 1.13 (0.54-2.35) | | ns | |
| Glucose (mg/dL) | 0.94 (0.80-1.11) | ns | | 1.01 (1.0-1.01) | | **0.020** | |
| Haemoglobin (g/dL) | 1.0 (0.99-1.0) | ns | | 0.90 (0.77-1.07) | | ns | |
| mean UL-PRx | 6.98 (1.89-40.95)* | **<0.001** | | 8.04 (1.26-51.08) | | **0.027** | |

CI = Confidence IntervalOR= Odds Ratio, ns= not significant results, ref= reference category. GCS = Glasgow Coma Score; CT = computed tomography; tSAH = traumatic Sub-Arachnoid Hemorrhage. *Note that the OR corresponds to a 0.1 unit change in the explanatory variable.
