# Supplementary material for: Cerebral autoregulation in traumatic brain injury: ultra-low-frequency pressure reactivity index and intracranial pressure across age groups
Source: Crit Care. 2024 Jan 23;28:33. doi: 10.1186/s13054-024-04814-5 (PMC10807228; doi:10.1186/s13054-024-04814-5)
Supplement: Supplementary file 6 — Additional file 6. Table S5. Multivariate logistic regression analysis in middle groups [file 13054_2024_4814_MOESM6_ESM.docx]

**Table S5. Multivariate logistic regression analysis in middle groups**

| **12-month mortality** | | | |
| --- | --- | --- | --- |
| **Model** | **AUC (95% CI)** | **AIC** | **Adjusted R^2^** |
| **IMPACT- Core** | 0.80 (0.71-0.89) | 137.68 | 0.22 |
| **IMPACT- Core + CT** | 0.84 (0.76-0.91) | 136.92 | 0.27 |
| **IMPACT- Core + CT + Lab** | 0.84 (0.78-0.91) | 139.05 | 0.28 |
| **IMPACT- Core + CT + Mean UL-PRx** | 0.89 (0.81-0.96) | 121.31 | 0.35 |
| **IMPACT- Core + CT + Lab + Mean UL-PRx** | 0.89 (0.82-0.97) | 123.78 | 0.36 |
| **12-month unfavorable outcome** | | | |
| **Model** | **AUC (95% CI)** | **AIC** | **Adjusted R^2^** |
| **IMPACT- Core** | 0.78 (0.73-0.87) | 176.50 | 0.22 |
| **IMPACT- Core + CT** | 0.86 (0.80-0.91) | 169.07 | 0.36 |
| **IMPACT- Core + CT + Lab** | 0.87 (0.81-0.92) | 166.37 | 0.38 |
| **IMPACT- Core + CT + Mean UL-PRx** | 0.86 (0.80-0.91) | 166.12 | 0.38 |
| **IMPACT- Core + CT + Lab + UL-PRx** | 0.87 (0.81-0.93) | 166.14 | 0.39 |

Middle group (159 patients, > 16 and < 70 y)

AUC= Area Under the Curve. CI = Confidence Interval. AIC= Akaike Information Criterion. p value < 0.001 for all the models.
