# Supplementary figures and images for: Cerebral autoregulation in traumatic brain injury: ultra-low-frequency pressure reactivity index and intracranial pressure across age groups
Source: Crit Care. 2024 Jan 23;28:33. doi: 10.1186/s13054-024-04814-5 (PMC10807228; doi:10.1186/s13054-024-04814-5)

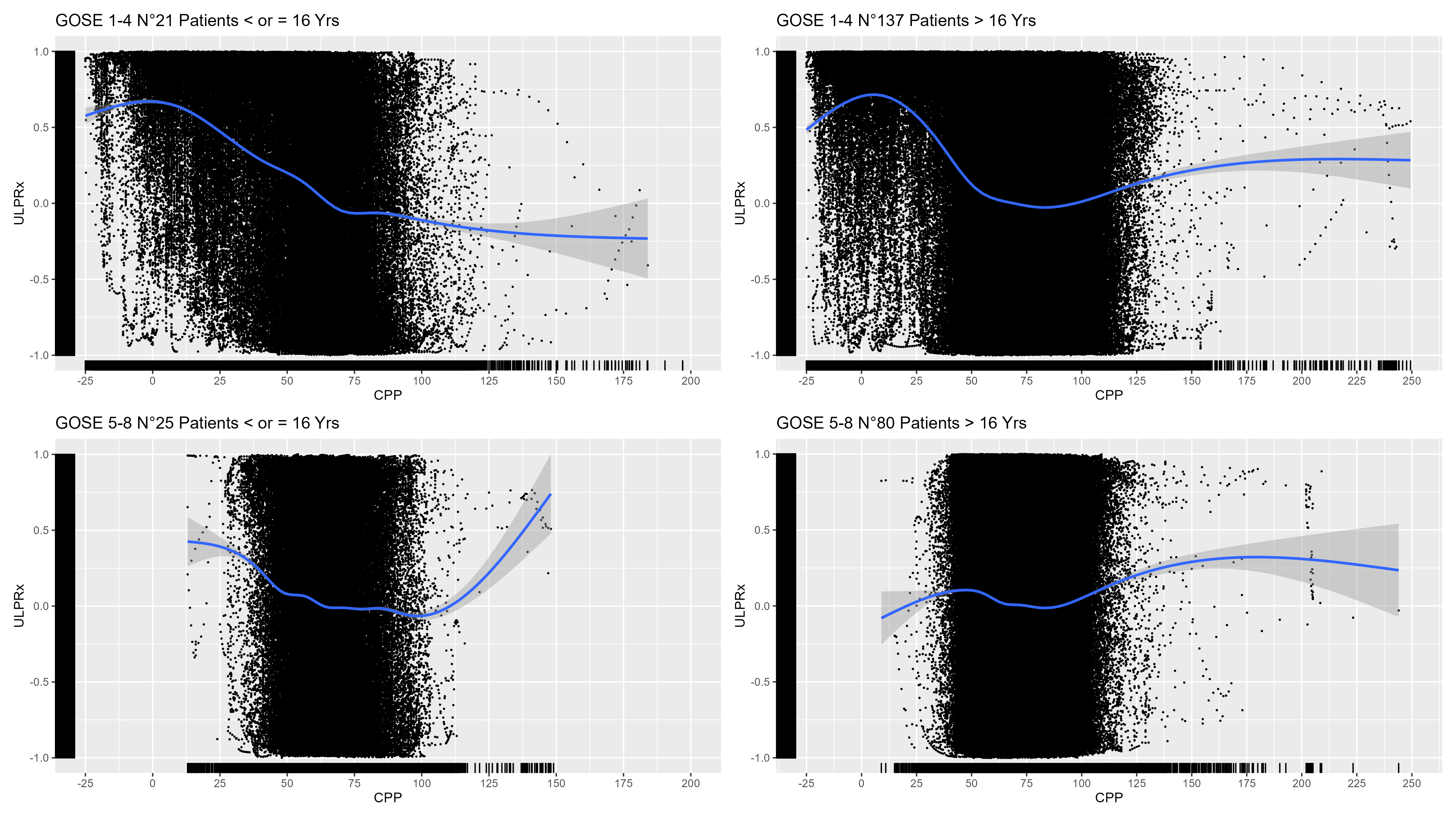

Supplement: Supplementary file 7 — Additional file 7. Figure S1. [file 13054_2024_4814_MOESM7_ESM.jpg]
